# Factors affecting implementation of perinatal mental health screening in women of refugee background

**DOI:** 10.1186/s13012-016-0515-2

**Published:** 2016-11-18

**Authors:** Nishani Nithianandan, Melanie Gibson-Helm, Jacquie McBride, Amanda Binny, Kylie M. Gray, Christine East, Jacqueline A. Boyle

**Affiliations:** 1 Monash Centre for Health Research and Implementation, School of Public Health and Preventive Medicine, Monash University, 43-51 Kanooka Grove, Clayton, 3168 Victoria Australia; 2 Monash Health Refugee Health and Wellbeing, Monash Health, Clayton, Victoria Australia; 3 Centre for Developmental Psychiatry and Psychology, Department of Psychiatry, School of Clinical Sciences, Monash University, Clayton, Victoria Australia; 4 Monash Women’s Maternity Services, Monash Health, Clayton, Victoria Australia; 5 School of Nursing and Midwifery, Monash University, Clayton, Victoria Australia

**Keywords:** Refugees, Pregnancy, Prenatal care, Mental health, Depression, Anxiety, Post-traumatic stress disorder, Perinatal, Health services research

## Abstract

**Background:**

For women of refugee background, the increased risk of mental illness associated with pregnancy is compounded by pre- and post-settlement stressors. In Australia, antenatal screening for depression and anxiety symptoms using the Edinburgh Postnatal Depression Scale is recommended for all women. Despite this, screening is not routinely implemented and little is known about barriers and enablers to implementation for women of refugee background.

**Methods:**

Semi-structured interviews were conducted with a range of health professionals (*n* = 28: midwives, obstetricians, perinatal mental health and refugee health experts, interpreters) and women of refugee background (*n* = 9). Themes generated from thematic analysis were examined in relation to the Theoretical Domains Framework and Cultural Competence Conceptual Framework, followed by identification of effective behaviour change techniques to address the barriers and enablers identified by participants. These techniques formed the basis of recommendations to inform sustainable implementation of screening and referral.

**Results:**

Almost all participants perceived perinatal mental health screening to be necessary and most recognised the importance of post-traumatic stress disorder (PTSD) screening. Barriers and enablers were identified and related to eight domains: knowledge, skills, professional roles, beliefs about capabilities and consequences, environmental context, social influences and behavioural regulation.

**Conclusions:**

This research clarifies *how* mental health screening may be integrated into routine antenatal care for women of refugee background, in order to improve provision of recommended care. These theory-informed recommendations include an inter-disciplinary approach, coordinating care within and across services, addition of PTSD screening, and effective communication with women.

## Background

Perinatal mental illness is a significant global problem, with postnatal depression prevalence ranging from 13 to 20% [1, 2]. Of women with postnatal depression, almost 40% are estimated to have developed symptoms during pregnancy [3]. Perinatal anxiety disorders may be as prevalent as depression [4], and higher levels of antenatal anxiety increase the risk of postnatal depression [5].

Pregnancy is a time of heightened vulnerability for the development or recurrence of mental illness [6] and women of refugee background are likely to be at greater risk than the general population given pre- and post-settlement stressors [6, 7]. Estimates of mental illness prevalence in general refugee populations vary, with systematic reviews reporting depression prevalence of 5–31% and post-traumatic stress disorder (PTSD) 9–31%, with evidence of significant comorbidity [8, 9]. The prevalence of *perinatal* mental illness in this demographic is poorly documented. Throughout this paper, the term ‘refugee background’ refers to women who self-report either a refugee or asylum seeker background. Refugees are persons with a well-founded fear of persecution, who are outside of their country of origin and unable or unwilling to return, while asylum seekers are persons seeking protection whose refugee status is unconfirmed [10].

Perinatal mental illness presents a major public health challenge, given its contribution to maternal morbidity and indirect mortality [6], adverse obstetric outcomes [11], and impaired psychological and physical development of infants and children [12, 13]. Partners’ quality of life and mental health may also be affected, and other children in the family may experience a greater risk of mental illness and adverse social and behavioural outcomes [6, 14, 15]. Thus, there is a clear rationale for antenatal screening to identify early symptoms and provide appropriate follow-up and management to prevent exacerbation of symptoms and improve outcomes. Moreover, the regular contact between health professionals (HPs) and women during pregnancy supports the rationale for integrating screening into routine antenatal care [16].

Australian clinical practice guidelines recommend routine antenatal assessment of (i) psychosocial risk factors and (ii) depression and anxiety symptoms using the Edinburgh Postnatal Depression Scale (EPDS), an extensively used and validated perinatal screening tool [6, 17].

However, antenatal screening is not routinely implemented at many hospitals [18], and little is known about how to integrate mental health screening into antenatal care. Barriers include lack of time, funding or follow-up infrastructure and inadequate training [6, 19]. Few enablers have been identified but include raising awareness amongst HPs, support from hospital management and development of follow-up pathways [19, 20].

Implementation is likely to be more complex for women of refugee background given their vulnerability and barriers to accessing health services such as lack of interpreters or healthcare literacy, Western medical models and stigma associated with mental illness [7, 21–23]. Previous studies of maternity care models with women of refugee background have not explored mental health screening [21, 22], system challenges or factors critical to success in rolling out a comprehensive screening and referral programme [24, 25].

Monash Health is located in south-east Melbourne in the Australian state of Victoria. It is one of the largest maternity service providers in Australia and also services a region with one of the largest resettled refugee populations in the country, up to 8.7% of the regional population [10, 26]. Importantly, a large proportion—40% over the last 10 years—of persons resettled under Australia’s Humanitarian Programme were women of child-bearing age [27]. Women self-reporting a refugee background are preferentially allocated to the Monash Health refugee antenatal clinic where possible. Psychosocial risk factor assessment, which aims to identify risk factors associated with perinatal mental illness such as past history of mental illness, current or past abuse, substance abuse and lack of social support [6], is routinely undertaken at Monash Health. However, screening for anxiety and depression symptoms is not undertaken at all, which is likely to lead to considerable under-recognition of women at risk of perinatal mental illness. This evidence-practice gap in antenatal care is widespread, with under a third of state public maternity hospitals reporting use of a psychosocial risk factor assessment tool and a quarter of hospitals reporting use of the EPDS [18]. This study aimed to (i) investigate barriers and enablers to implementing evidence-based, nationally recommended perinatal mental health screening and (ii) inform sustainable implementation of a screening and referral programme, in women of refugee background.

## Methods

### Study design

Qualitative research methods were deemed most appropriate to elicit in-depth stakeholder perspectives [28]. Semi-structured interviews were selected as they provide some guidance, while allowing the interviewer to be responsive to participants, empowering stakeholders to explore issues they identify as significant and providing an environment conducive to working with interpreters [29, 30]. This decision was further supported as many interviewed women described their own struggles with mental illness, which may not have been volunteered in a group setting [29]. While in-person interviews afford greater opportunity to build rapport and observe body language, telephone interviews can be more convenient and less intimidating [29]. Hence, participants were offered both options.

### Materials

Preliminary consultations with stakeholders suggested three areas of focus for the interview guide: (i) rationale for screening, (ii) acceptability of the screening tool and (iii) barriers and enablers to implementation of screening and referral. Based on these consultations, the interview guide (Appendix) was developed by three of the authors and pilot-tested with two independent researchers for feedback regarding open-ended enquiry, appropriate use of probes, and interview length. Once interviews with participants commenced, an iterative approach was adopted in which the interview guide was revised based on key ideas raised in previous interviews. For example, PTSD screening was discussed by one participant and then incorporated into the interview guide for subsequent interviews. The first named author and each participant jointly determined to what extent topics were explored, depending on the interviewee’s role. The EPDS was brought as a prompt for community representative (CR) interviews.

### Recruitment

Participants were approached during April–July 2015. HPs and researchers with specialist knowledge in perinatal mental health, refugee health or both, or who were involved in the Monash Health refugee antenatal clinic were purposively recruited. A broad range of stakeholders was sought because the topic intersects three distinct areas of healthcare: refugee health, pregnancy care and mental health and given the multidisciplinary nature of pregnancy care. All CRs and other HPs were recruited through snowballing techniques, which is useful in accessing ‘hard-to-reach’ populations [30]. CRs with a current or previous pregnancy and a refugee or asylum seeker background were recruited. Recruitment of CRs was assisted by a bicultural worker and Adult Multicultural Education Services volunteers, who introduced the study to potential participants and gauged interest before recommending them to the first named author. Accredited interpreters were arranged for non-English-speaking CRs. Recruitment continued until data saturation was achieved.

### Participant characteristics

The study sample included 28 HPs/interpreters and 9 CRs from diverse ethnic backgrounds (Tables 1 and 2). Most individuals who were approached consented to participate in interviews (37/42). Two obstetricians failed to respond to interview requests, and three CRs were lost to contact. Of the CRs, four required interpreters.

**Table 1:** Number of participants according to role

Role		Number of participants (*n* = 37)
Staff	Midwives	5
	Obstetricians	6
	Maternal and child health nurses	2
	Perinatal mental health nurses	2
	Perinatal and infant psychiatrist	1
	Perinatal mental health expert	1
	Maternity general practice liaison officer	1
	Community mental health team leader	1
	Refugee health nurse^a^	1
	Refugee health experts^b^	3
	Bicultural worker	1
	Interpreters	4
Community representatives		9

Many participants occupied multiple roles; the most relevant role is listed

^a^A nurse or midwife trained to assess, educate, refer and coordinate care for people of refugee or asylum seeker background

^b^Someone with extensive research or clinical experience with women of refugee background

**Table 2:** Ethnic backgrounds of community representatives, interpreters and bicultural worker

Ethnic background	Number of participants (*n* = 14)
Burmese (Rohingya)	2
Afghan	5
Sudanese	1
Iranian	3
Iraqi	1
Sri Lankan (Tamil)	1
Indian	1

### Data collection and analysis

All interviews were audio-taped and transcribed verbatim by a professional transcription service. Transcripts were verified by the first named author, and participants were invited to review their transcripts. De-identified transcripts were imported into NVivo (Version 10) and thematic and inductive (‘data-driven’) analysis was conducted, involving data coding, reviewing the list of codes, organising codes within themes, revising themes against codes for inclusiveness and ongoing refinement of the thematic map [29]. Thematic analysis was conducted prior to identifying best fit theoretical framework(s) from the literature to avoid coding data to fit into a pre-existing framework [29]. Analysis was undertaken independently by two of the authors, with constructed themes compared for inconsistencies. This, along with researcher reflexivity, documentation of field notes and key analytic decisions and inclusion of representative quotes, contributed to research credibility, dependability and confirmability and thus analytic rigour [28].

Thematic analysis yielded a set of themes, which were closely examined in relation to the Theoretical Domains Framework (TDF) [31] and domain constructs to better understand behavioural determinants. The two analysts independently determined key domains to be those that described the barriers and enablers within the themes at a theoretical level. Interpretation of data was enhanced by the Cultural Competence Conceptual Framework (CCCF), which adds a cultural competence lens to the process of understanding behaviours and intervention design using the TDF. The CCCF describes three stages to achieving cultural competence: critical awareness and cultural knowledge, skills development and organisational support for multicultural practice [32]. As cultural competence was expected to be a key component of an appropriate screening programme in women of refugee background, the CCCF was deemed to provide an essential perspective not addressed by the TDF.

Of relevance to introducing a screening and referral programme, theory-informed, evidence-based, healthcare interventions are more effective than those lacking a theoretical foundation [31, 33, 34]. Both the TDF and CCCF are well-validated, successfully applied frameworks that encompass individual and organisational influences. Previous implementation studies have been informed by the mapping of TDF domains onto behaviour change techniques at an individual or organisational level [33–35] or have used two theoretical frameworks simultaneously to plan an optimal intervention [36].

In developing recommendations for a screening programme (Table 3), our understanding of *what* behaviours to target and *how* to effectively do so was informed by Michie et al.’s matrix, which maps theoretical domains (i.e. behavioural determinants) to effective behaviour change techniques using an expert consensus process [34]. After identifying potential behaviour change techniques for each domain, the research team and other members of a broader steering committee within the Monash Women’s Maternity and Monash Health Refugee Health and Wellbeing services used their multidisciplinary experience to select the techniques most relevant to the setting. The same group developed recommendations (Table 3, columns 3 and 4) by applying these techniques to specifically address the barriers and enhance the enablers identified by participants.

## Results

### Key findings

**Fig. 1:**
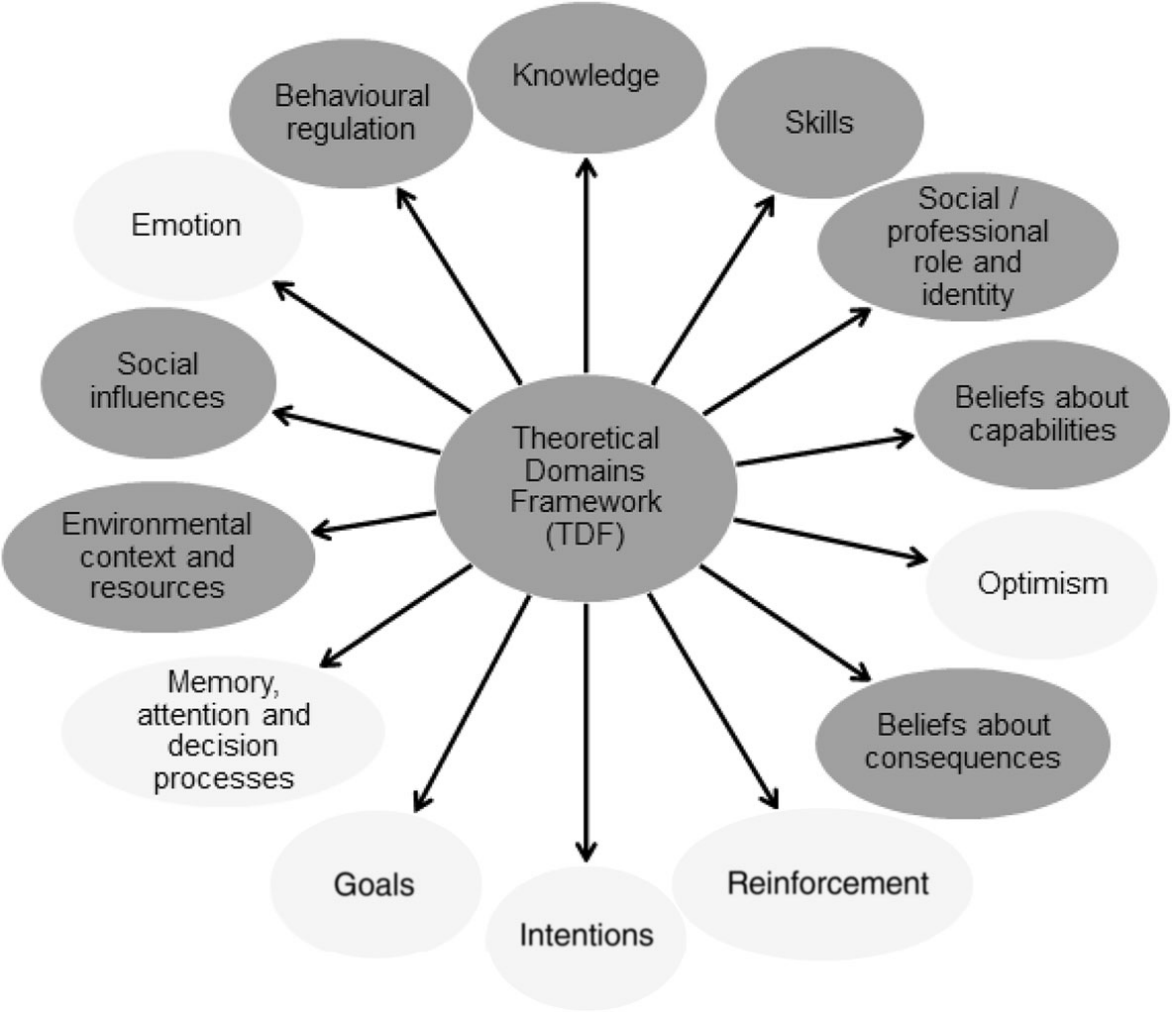
*Theoretical Domains Framework* (*dark grey circles* represent domains relevant to key findings)

Key findings are presented within eight key TDF domains (Fig. 1), which described the barriers and enablers within the themes at a theoretical level. The remaining six domains of the TDF were not relevant to the barriers and enablers.

#### Knowledge

Almost all participants perceived the need for, and understood the rationale behind, routine antenatal mental health screening:

…it is very very necessary because it's not only affects the woman herself but the whole family… I know it takes time and it needs money…but in order to make a better community we need to look into people's problems more deeply. Especially women because they are the core of the family…if they fall, everybody falls. Children, and husbands and partners you know everybody will be affected. (ID 4; interpreter)

One participant was ambivalent, citing the rigidity of universal screening, false positives and negatives, and inappropriate management. Few HPs volunteered knowledge of clinical practice guidelines regarding antenatal screening, and knowledge and experience of EPDS administration was variable.

While HPs commonly believed women of refugee background had a ‘lack of understanding of mental health’ (ID 24; HP), most CRs demonstrated some knowledge of mental health often drawn from their experience of symptoms (articulated as worry, crying, difficulty focusing, self-blame, ‘pressure on brain’, ‘going mental’, feeling depressed or sad) or mental health services (MHS). Many CRs and some HPs spoke about mental health in a broader psychosocial context. One interpreter insisted that mental illness was common but *perinatal* mental illness rare amongst Burmese women. Many HPs and CRs recommended community mental health education.

Multiple HPs explored the cumulative impacts of trauma, pregnancy and post-settlement stressors, and both HPs and CRs anticipated high prevalence of mental illness. Most women from culturally and linguistically diverse backgrounds described previous trauma impacting the foetus or leading to women not sleeping, eating or taking medication whereas HPs explored exacerbation of PTSD symptoms in pregnancy and its:

…significant impact on how women cope with pregnancy in terms of antenatal appointments… physical examinations… (ID 22; HP)

While most participants perceived PTSD screening as important, most lacked knowledge of trauma screening tools.

#### Skills

Most HPs advocated for staff ‘training on not only how to screen but… what you’re screening for and what does it mean and what do you do with that information?’ (ID 9; HP) Some also recommended training in cultural competence and identification and prioritisation of refugee health needs:

…I don't think there's a lot of understanding with the midwives about, um, refugee health needs… the problem is they don't have a lot of background in this. (ID 27; HP)

This perceived lack of understanding was thought to result in incomplete referrals, which failed to specify required services or the reason for referral beyond ‘refugee background’.

#### Social/professional role and identity

All HPs asserted that they had a role to play in the implementation of screening, referral or management. Many described refugee health nurses acting as a ‘go-to’ person for staff and supporting women to access referrals:

…a face to face introduction to somebody in the Refugee Health Service such as [refugee health nurse] makes a huge difference to a woman’s likelihood of accepting a referral to a service… (ID 10; HP)

Bicultural workers or community volunteers were widely perceived to improve cultural appropriateness of screening and follow-up. A few participants discussed the importance of perinatal mental health nurses in advising staff regarding women who are suicidal, at risk of self-harm or who have a significant history of mental illness and to facilitate appropriate triaging.

Midwives perceived their involvement throughout perinatal care but:

…our main focus would be identifying [cases] and referrals… (ID 14; HP)

Obstetricians advised that midwives administer screening—citing shorter obstetric appointments occurring later in pregnancy or the fact that midwives already undertook psychosocial risk factor assessment—though they outlined their responsibility to support referrals and monitor patients’ progress.

A few HPs recommended involving social work and general practitioners. Participants from the Monash Health Refugee Health and Wellbeing service and community MHS felt that they had roles in providing referral pathways, triaging referrals and outreach support.

Participants desired clarity around roles within and between health services. A few HPs urged continuity and information sharing throughout the peripartum period to prevent:

…miscommunication and fragmentation to occur between GP [general practitioner], midwife, maybe an obstetrician if they get involved, Maternal and Child Health…and then all these other services; people fall through the gaps. (ID 25; HP)

#### Beliefs about capabilities

HPs expressed concern about when to refer, and who to refer to, and proposed that staff have access to telephone numbers and a support person onsite.

…a lot of times when I’ve had to deal with that it’s been…a big learning curve at the time … it’s not streamlined and it’s not efficient. It takes me a long time…to resource what I’m supposed to do about this kind of thing… (ID 14; HP)

Some HPs suggested engaging staff in order to engage women to complete screening:

…if we’re able to communicate the difference that this has the potential to make to women in their care they’re far more likely to champion it…If we engage the staff they will engage the women. (ID 10; HP)

While HPs varied in their suggested approaches to PTSD screening, many urged a cautious, sensitive approach and acknowledged their lack of expertise:

I don’t know how I would personally ask somebody if they’d experienced trauma. (ID 13; HP)

#### Beliefs about consequences

Both CRs and HPs identified stigma and apprehension around interpreters breaking confidentiality as potential barriers to women disclosing symptoms of mental illness and accessing MHS. Many CRs remarked that there may be differences between their own accepting attitudes towards mental illness and their communities’ attitudes. A few CRs noted that their confidentiality concerns were allayed if they trusted the interpreter’s professionalism.

CRs and interpreters generally perceived the EPDS as appropriate and easy to understand with an accurate interpreter:

…it’s very appropriate to ask the refugee women like this kind of questions… (ID 36; CR)

Some HPs described the EPDS as straightforward and the best available screening tool; however, other HPs were unsure about its appropriateness, citing different cross-cultural understandings or manifestations of mental illness or noting that the EPDS overlooks trauma.

Normalising screening by ‘talking about it…at previous appointments’ (ID 8; HP), describing it as routine pregnancy care, sensitively framing MHS and providing follow-up care that women perceive to be useful to themselves or their children were identified as enablers to screening and referral uptake. Many CRs valued being able to express their feelings at counselling sessions (‘I’ve emptied myself…’ (ID 34; CR)) or desired provision of practical strategies, or both:

to be helpful…and more culturally suitable…it needs to give them some more practical advice…how to control their anxiety. (ID 4; interpreter)

Multiple CRs and HPs anticipated that the culturally competent, multidisciplinary service offered by the Monash Health Refugee Health and Wellbeing service would overcome many barriers to access.

#### Environmental context and resources

Access to accurate, on-site interpreters was unanimously discussed, with phone interpreters deemed inappropriate for mental health consultations. Several HPs perceived interpreters’ skill as variable and a few suggested providing standardised instructions regarding EPDS translation. Many HPs and CRs advised having a female HP/interpreter to improve cultural appropriateness, particularly for women with traumatic histories:

…with a female interpreter, we feel safe and we feel comfortable so everything that we wanted to express…we can say it easily… (ID 34; CR)

While many HPs identified the importance of translated screening tools, literal translation of the EPDS was widely considered inadequate and HPs advised back translation and gaining community input to better achieve cultural equivalence.

Most HPs identified lack of time as a barrier:

How much extra time do you need to allocate when you get …a high…a positive?…you need to have the capacity within your system to manage it if you've got someone who's suicidal… (ID 23; HP)

Participants were divided between screening as early as possible (e.g. most CRs) to facilitate earlier referral and waiting until a less time-pressured appointment when sufficient rapport had been built. Most obstetricians felt the first hospital visit with the midwife was sufficiently long; however, midwives insisted this visit was already very full. Some HPs suggested the second visit and advised repeating screening in third trimester or abstaining from screening if women were known to be linked-in with services:

…have some clinical discretion so that if you have a woman who presents with a really flat affect or is very teary or discloses spontaneously…that’s the appropriate time to screen… (ID 10; HP)

Multiple HPs perceived screening as ‘step one’ (ID 24; HP) and questioned the capacity and sustainability of MHS to accept referrals*.* A few urged initial consultation with and mapping of community MHS. Other common enablers included a private setting for screening (i.e. not the waiting room) and educating and assisting with transport.

#### Social influences

All CRs described loss of social support contributing to poor wellbeing. HPs and CRs considered family members to be protective against or contributors to mental illness or barriers to honest disclosure and help-seeking. A few HPs encouraged:

…making sure that the husband or other family members are okay with…the woman attending this type of referral… (ID 19; HP)

All CRs and some HPs regarded continuity of carers as critical to build trust, improve symptom monitoring and encourage disclosure:

…every day my doctor was changed I couldn’t make a relationship with my…doctor. (ID 32; CR)

Several HPs prioritised having a ‘go-to’ person (i.e. refugee health nurse, senior staff, psychiatry liaison) for the staff who were administering screening. Infrequent suggestions included debrief opportunities, team meetings and support from hospital leadership.

#### Behavioural regulation

Factors that may facilitate changed practice at an individual or organisational level were identified by HPs. Some HPs identified the need for immediate follow-up for positive responses related to suicidal thoughts and long-term follow-up processes:

…the most important thing is follow-up… that if someone doesn’t turn up for their appointment, that that’s flagged…and that they’re contacted. (ID 2; HP)

Most HPs discussed the need for clear referral guidelines (e.g. displayed as flowcharts in clinic rooms), and suggested that:

…those referral pathways are pretty simple if they’re clearly articulated… (ID 28; HP)

Some HPs perceived that multiple referral pathways could be advantageous for different women’s needs, whereas others anticipated that multiple pathways could be confusing for staff. Some participants anticipated improved referral uptake if follow-up care was provided on-site. Other critical success factors included minimising time between referral and follow-up, clear documentation, communication between services and feedback mechanisms to confirm receipt of referral.

## Discussion

### Summary of key findings

This study systematically defines barriers and enablers to implementation of perinatal mental health screening and referral for women of refugee background. Participants overwhelmingly recognised the need for mental health screening and PTSD screening. Factors affecting implementation identified by HPs included staff training needs, inter-disciplinary roles to support referral and clearly communicated, robust referral pathways. CRs prioritised continuity of care, female interpreters and HPs, social support and useful follow-up care. Key environmental considerations included availability of in-person interpreters, rigorously translated EPDS versions, time constraints and capacity of MHS.

Effective behaviour change techniques for each of the eight domains (i.e. behavioural determinants) were identified from Michie et al.’s matrix, which maps theoretical domains to behaviour change techniques. The multidisciplinary research team, including members from the Monash Women’s Maternity and Monash Health Refugee Health and Wellbeing services, then selected the most relevant techniques to this setting, developed recommendations (Table 3) and compared these recommendations with the current literature.

### Context and implications for clinical practice

#### Perceived need for perinatal mental health and PTSD screening

The perceived necessity of perinatal mental health screening is an indicator of system readiness for change and is associated with a greater likelihood of effective implementation [37]. The perceived need to screen for PTSD symptoms in routine antenatal care of women of refugee background has not previously been reported, and specific PTSD screening may address the concern of a few HPs that the EPDS overlooks trauma. The varied opinions as to *how* to execute PTSD screening may reflect participants’ concerns about exacerbation of symptoms if screening is inappropriately administered or differing success with screening methods. This highlights the need for careful selection of appropriate perinatal mental health and PTSD screening methods and training for HPs in sensitive administration (*Beliefs about capabilities*, Table 3).

**Table 3:** Recommendations for implementation of perinatal mental health screening in women of refugee background

Behavioural determinant	Behavioural change techniques^a^	Examples to support health professionals (HPs)	Examples to support women
Knowledge	Information regarding behaviour, outcome	Provide information for HPs regarding rationale for screening; clinical guidelines and evidence-practice gap; appropriate EPDS administration, scoring and actions; and PTSD screening	Provide information (e.g. culturally appropriate group sessions, translated printed materials) at earlier appointments about perinatal mental illness, routine screening, and MHS
Skills	Goal/target specified: behaviour or outcome Increasing skills: problem solving, decision making, goal setting Rehearsal of relevant skills	Organisation to set target of routine screening; individual HPs to set targets for skills attainment Provide training for HPs regarding identification and prioritisation of refugee health needs; appropriate use, scoring and actions to EPDS; and cultural competence (including approach to mental health and managing family members) Provide opportunities to practise skills	
Social/professional role and identity	Social processes of encouragement, pressure, support	Involve refugee health nurse, bicultural worker, perinatal mental health nurse and senior staff to support referral Balance inter-disciplinary approach with clear delineation of roles Ensure clear communication between antenatal and postnatal services and identify women already receiving mental health care	
Beliefs about capabilities	Increasing skills: problem solving, decision making, goal setting Social processes of encouragement, pressure, support	Provide training for HPs (i.e. sensitive administration of trauma screening tool, management of women at risk of suicide or self-harm) Engage staff by communicating the rationale for screening and benefits for women	
Beliefs about consequences	Persuasive communication Information regarding behaviour, outcome	Provide information for mental HPs regarding the provision of refugee appropriate mental health care (e.g. practical advice about managing symptoms)	HPs to normalise screening; provide culturally appropriate mental health information at earlier appointments; manage expectations regarding referrals; and communicate professionalism of interpreters and usefulness of follow-up mental health care
Environmental context and resources	Environmental changes (e.g. objects to facilitate behaviour)	Select the most appropriate time(s) to screen with input from HPs administering the EPDS (e.g. second antenatal visit and again in third trimester). Allow HPs discretion to screen earlier or later or to forgo screening if guided by MHS already involved in care Management to work with HPs to allow appropriate appointment length and flexibility to manage disclosures and make immediate referrals Map MHS in the area and confirm capacity and sustainability of services prior to implementation	Incorporate rigorously translated screening tools into routine maternity care Provide skilled, onsite, female interpreters for common refugee languages and standardised instructions for appropriate EPDS translation Screen in a private setting Provide advice around transport
Social influences	Social processes of encouragement, pressure, support	Ensure a ‘go-to’ or support person for HPs (e.g. refugee health nurse, senior staff, psychiatry liaison), regular team meetings and debrief opportunities	Ensure continuity of care Include referral pathways to social work, women’s groups and language services HPs to explain to family members what screening and potential follow-up involves; however screening to be undertaken privately
Behavioural regulation^b^	Planning, implementation Prompts, triggers, cues	Establish robust referral pathways, feedback mechanisms to confirm receipt of referrals, communication channels between services, and clear documentation at all stages of pathways Clearly communicate pathways (e.g. flowcharts) and contact numbers for to HPs	Establish various pathways for different needs while minimising referral points Use on-site services where possible (e.g. social worker)

*HP* health professional, *EPDS* Edinburgh Postnatal Depression Scale, *MHS* mental health services

^a^Behaviour change techniques recommended by Michie et al.’s matrix [34] and deemed to be relevant to this setting

^b^Behaviour change techniques listed for *Action planning* in Michie et al.’s matrix [34] have been used here interchangeably with *Behavioural regulation* given the likeness between the domains

#### Inter-disciplinary approach

The clear differences in roles attributed to each HP group and the recognised importance of refugee health nurse and perinatal mental health nurse roles uniquely highlight inter-professional relationships as important support structures for HPs involved in implementation [38] (*Social/professional role and identity*, Table 3). The perceived role of bicultural workers in educating and supporting women of refugee background is supported by the literature [21, 25] and by the CCCF, which asserts that individuals’ awareness and knowledge alone are insufficient to result in culturally competent environments and organisations must also adapt services to be accessible to culturally and linguistically diverse patients [32]. That all HPs asserted a role at one or more stages of screening, referral and management confirms the need for an inter-disciplinary approach in this setting.

Inter-disciplinary collaboration involves pursuing common goals, shared decision-making and planning and open communication between HPs and services [38] to ensure clarity around roles and appropriate, timely referrals and to prevent fragmentation of care. An example of shared planning is liaison with psychiatry departments and community MHS to assess their capacity to absorb referrals in the long-term (*Environmental context and resources*). Low rates of referral to specialist MHS have been found in the UK (1–3% of women screened) and New South Wales, Australia, [6, 39] with women requiring less extensive care managed by general practitioners; however, referral rates may be higher for women of refugee background. Evaluation of local implementation efforts including referral rates would inform scaling-up of implementation. Shared decision-making is vital in determining optimal timing of screening administration, which is poorly covered in the literature. If midwives are to administer screening, recognising their perception of when it is practical to screen is likely to facilitate ownership and adoption of screening (*Environmental context and resources*, Table 3). In keeping with the ‘organisational support’ domain of the CCCF [32], health service managers need to work with HPs to allow flexibility to manage disclosures and make immediate referrals.

Inter-disciplinary collaboration also requires flexibility in sharing professional responsibilities to improve clinical care [38]. Not all HPs volunteered a direct role in screening. For instance, obstetricians recommended that midwives administer screening while they supported the referral and follow-up process; however, all HPs involved in antenatal care should receive training to appropriately fulfil their role whether they are directly involved in administering screening or not (*Skills*, Table 3).

#### Effective communication with women

Achieving cultural equivalence in EPDS translations and having accurate, female interpreters is consistent with the literature [21, 24, 40, 41]. Results from this study add that female interpreters are a priority specifically for mental health conversations to encourage disclosure from women with traumatic backgrounds. Translations of the EPDS are freely available for most refugee languages spoken in the local area [42]. Institutional investment, such as incorporating translated screening tools into routine maternity care and ensuring availability of onsite interpreters for common languages of resettled refugees, reflects the organisation’s commitment to sustainable implementation [37] and is essential to a culturally competent service [32] (*Environmental context and resources*, Table 3).

Beliefs about consequences are known to affect implementation success [37] and persuasive communication and provision of information to HPs, and women are required to enhance and address these positive and negative beliefs respectively (*Beliefs about consequences*, Table 3). Some HP concerns regarding EPDS administration in this population are consistent with other research [24]. However, the positive perceptions of the EPDS by CRs and interpreters and their understanding of the impacts of perinatal mental illness contrast with a recent study, which described women’s lack of understanding of EPDS items and concepts, and postulated that women of refugee background lacked a framework for understanding the rationale behind EPDS administration [24].

These differences may be attributed to greater exploration of CR understanding of the rationale for screening in this study; some CRs having experienced symptoms of mental illness or mental health care or differences in participant demographics (predominantly African compared with mostly Asian countries in this study), study design (focus groups and surveys compared with interviews) or familiarity with the EPDS. Further research is needed to investigate cross-cultural understandings of each EPDS item with women from key refugee communities.

Consistent with the literature [23, 40], there was some evidence that women of refugee background may use different words to articulate perinatal depressive symptoms or may misunderstand the ‘perinatal onset’ qualifier. Thus, when explaining the EPDS and undertaking diagnostic assessment, HPs need to consider women’s different expressions for perinatal depression. However, many CRs also noted their own accepting attitudes towards mental illness differed from their communities’, possibly stemming from personal experiences of mental illness and MHS, education or supportive family and friends. This challenges the notion of stigma and different cross-cultural understandings as insurmountable barriers. Along with CR understanding of the rationale, this supports the provision of culturally appropriate information about mental health and routine screening at earlier appointments to increase acceptance of later screening and follow-up (*Knowledge* and *Beliefs about consequences*, Table 3).

Normalising screening, managing expectations regarding referral(s) and communicating the professionalism of interpreters and usefulness of follow-up care are recommended to allay women’s concerns and encourage engagement with services (*Beliefs about consequences*, Table 3). This study contributes to an under-researched area [43] and suggests that women of refugee background perceive ‘useful’ MHS to offer practical advice and opportunities to express emotions, thus providing valuable feedback for mental HPs.

### Limitations and strengths

While indistinct boundaries between some TDF domains may be considered a limitation, this reflects the often multiple determinants of behaviours and confirms that behaviour change requires a multi-faceted approach. A smaller number of CRs were recruited compared with HPs; however, data saturation was achieved to fulfil the study aims. While many of the CRs had accessed maternity services since coming to Australia (some of whom were currently pregnant) and these experiences informed their responses, this study did not specifically recruit CRs currently attending a maternity service. Current service users may identify additional barriers or enablers from their experience. To address this, recommendations from this study have informed a pilot programme designed by a broad steering committee and evaluation will include focus groups with women and health professionals. Strengths of this study include the range of participants interviewed, community consultation, rigorous study design and use of two generalizable, applied theoretical frameworks.

## Conclusions

While clinical guidelines recommend integrating screening into routine antenatal care, this formative research is the first to clarify *how* integration might be achieved for women of refugee background. This study reveals a prevailing attitude amongst stakeholders that perinatal mental health and PTSD screening is necessary in this population. Findings emphasise the importance of adopting an inter-disciplinary approach to implementation and facilitating effective communication with women and between and within health services. This research provides clear evidence around barriers and enablers, and theory-based recommendations to inform implementation of perinatal mental health screening and referral for women of refugee background and ultimately improve provision of recommended antenatal care.

## Appendix

### Intervention schedule

1. Can you tell me about your work/yourself?
2. Have you had any experience with mental health screening programs or screening tools?
3. How necessary do you think mental health screening is?
4. How much do you know about the EPDS?
  - Do you think the EPDS is an appropriate questionnaire to assess mental health in this population?
5. What do you understand about the role of trauma in affecting pregnancy?
  - What do you think about screening for trauma in pregnancy?
6. In your opinion, when during a woman’s pregnancy is the most appropriate time to conduct mental health screening?
7. Can you think of any challenges to setting up screening at the antenatal clinic and its sustainability (i.e. factors that would undermine screening over the long-term)?
  - How might those challenges be overcome?
8. What factors might help screening to be as effective as possible?
9. How might we improve the cultural appropriateness of screening? (How can we make screening more culturally sensitive?)
10. Can you think of any challenges to the referral process?
11. What do you think critical success factors for a referral process would be?
12. What barriers might exist to women accepting follow-up care at the Refugee Health and Wellbeing Service? (Why might women not accept follow-up care at the Refugee Health and Wellbeing Service?)
13. How could the refugee antenatal clinic or the Refugee Health and Wellbeing Service support women to access follow-up care?
14. Do you see your organisation as having a role in the implementation of screening and referral, and if so what role?
15. Is there anything else you would like to add?
  - Can you recommend anyone else we should speak to regarding implementation?

## Abbreviations

CCCF: Cultural Competence Conceptual Framework
CR: Community representative
EPDS: Edinburgh Postnatal Depression Scale
HP: Health professional
MHS: Mental health services
PTSD: Post-traumatic stress disorder
TDF: Theoretical Domains Framework
